# Intrahepatic Cholangiocarcinoma With Lung Metastasis in a 29-Year-Old Male Patient: A Case Report

**DOI:** 10.7759/cureus.39787

**Published:** 2023-05-31

**Authors:** Marco A Calle Prado, Maria F Casanova Rivera, Diego A Vasquez Cedeño

**Affiliations:** 1 Faculty of Medicine, Universidad Católica de Santiago de Guayaquil, Guayaquil, ECU

**Keywords:** cholangiocarcinoma, aggressive neoplasm, poor outcomes, metastatic cholangiocarcinoma, intrahepatic cholangiocarcinoma

## Abstract

Cholangiocarcinoma (CCA) is an uncommon biliary neoplasm that is more frequent in male patients. CCA is categorized into intrahepatic cholangiocarcinoma (iCCA) and extrahepatic cholangiocarcinoma (eCCA) associated with the anatomical origin location. The clinical presentation is non-specific and varies depending on the origin, iCCA is generally asymptomatic until advanced disease is present therefore this neoplasm presents a poor prognosis with a survival rate of two years. We present a case of iCCA with lung metastasis in a 29-year-old male patient with no risk factors for this malignancy.

## Introduction

Cholangiocarcinoma (CCA) is a rare malignancy of the biliary tree and represents 3% of gastrointestinal neoplasms [1,2]. CCAs have been classified according to their anatomical site of origin in intrahepatic (iCCA) and extrahepatic (eCCA), eCCAs have been sub-classified in perihilar and distal, 50% of CCAs arise in the perihilar region, 40% in the distal region, and 10% in the intrahepatic region [2,3]. The peak of incidence is the seventh decade of life with male predominance [4]. In most cases the cause leading to CCA can be traced to their chronic history of any of the risk factors including primary sclerosing cholangitis, choledochal cysts, chronic intrahepatic stone disease, chronic liver disease in the form of cirrhosis or chronic viral hepatitis are also being recognized; the strongest risk factors include non-alcoholic fatty liver disease and cholelithiasis associated with chronic inflammation. However, in most cases, no cause can be established [4-6].

The clinical manifestations of CCA are often subtle and depend if it is iCCA or eCCA. iCCA diagnosis is often incidental and occurs proximal to the second-order bile ducts which are the next largest branches or ducts of the biliary system which join to form or empty into the main hepatic bile duct, so symptoms of biliary obstruction are much less common compared with eCCA [7-9]. Jaundice is not frequent in iCCA, the symptoms are non-specific such as abdominal pain, fatigue, malaise, nausea, night sweats, and weight loss. Given these general and vague symptoms, iCCA is diagnosed at an advanced stage [8,10,11]. Conversely, eCCA most often present with jaundice, pruritus, clay-colored stools, tea-colored urine, and occasional right upper quadrant pain [7,8].

CCA has a poor prognosis with a median survival of two years [4,10]. Five-year survival remains low even with resection and ranges from 10 to 49% [7]. iCCA arising in non-cirrhotic livers has a poorer prognosis [11].

## Case presentation

A 29-year-old Ecuadorian man who had no previous medical history of diseases and denied family history of malignancies. The patient was admitted to the hospital for chief complaints of fatigue, malaise, and vomiting. Physical examination was unremarkable. Laboratory data are summarized in Table 1. His serum CA (carbohydrate antigen) 19-9, carcinoembryonic antigen (CEA), CA 72-4, liver kidney microsomal type 1 antibody (anti-LKM-1), antinuclear antibody (ANA), and soluble liver antigen (SLA) were within normal limits. He had negative serology for hepatitis B and C.

**Table 1:** Initial laboratory investigation ALT: alanine aminotransferase AST: aspartate aminotransferase GGT: gamma-glutamyl transpeptidase ALP: alkaline phosphatase

Laboratory test	Normal range	Result
White blood cells	4.10-10.10 10x9/L	9.9 10x9/L
Hemoglobin	12.9-16.7 g/dL	15.9 g/dL
Mean corpuscular volume	80.8-94.1 fL	90 fL
Platelet count	153-328 10x9/L	261 10x9/L
ALT	21-72 U/L	62 U/L
AST	17-59 U/L	67 U/L
GGT	15-73 U/L	687 U/L
ALP	38.0-126.0 U/L	225 U/L
Albumin level	3.5-5.0 g/dL	5 g/dL
Total bilirubin	0.2-1.3 mg/dL	1.10 mg/dL

**Figure 1:**
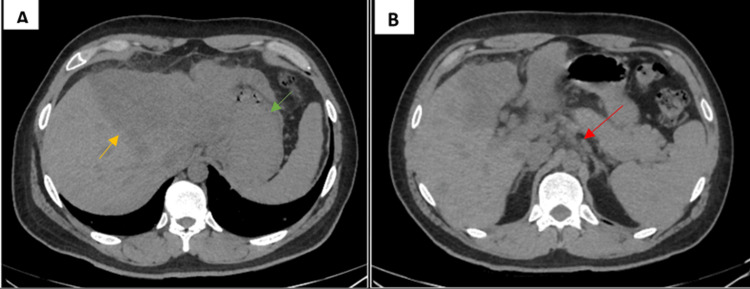
Abdominal CT demonstrated a low-density mass, involving the left hepatic lobe (green arrow) and medial segment of the right lobe (yellow arrow). B. The tumor exhibited celiac trunk adenopathies (red arrow). CT: computed tomography

Abdominal computed tomography (CT) scan with and without intravenous contrast was obtained and showed irregular liver contours with a space-occupying lesion with indistinct borders, which has a low density in the portal phase with heterogeneous enhancements postcontrast, with a greater axial diameter of 19 cm, involves the entire left hepatic lobe and partially the medial segments of the right lobe associated with low-density nodular images in the portal phase with ring enhancement of 20 mm, 11 mm, 10 mm as well as adenopathies in the course of the celiac trunk (Figure 1).

**Figure 2:**
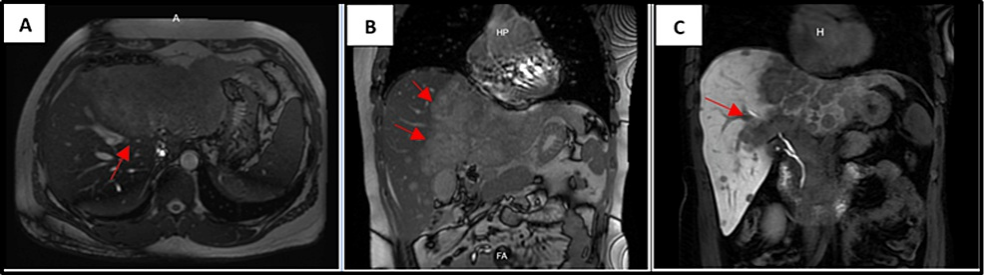
MRI demonstrated an irregular and heterogeneous mass (A). The mass showed multiple multisegmented nodular lesions in the right lobe (B). Biliary tree infiltration (C). MRI: magnetic resonance imaging

Abdominal magnetic resonance imaging (MRI) scan with and without contrast revealed a solid, expansive mass of approximately 20 x 12 cm that involves almost the entire left hepatic lobe and partially the medial segments of the right lobe, hyperintense on T2 with hypointense areas on T1 and a pseudocapsule on its periphery with peripheral enhancement in the early post-contrast phase, the mass causes irregularity of the hepatic contour associated with the presence of multiple nodular images inside, it is also associated with multiple multisegmented nodular lesions in the right lobe measuring 21 mm, 12 mm, 10 mm with ring enhancement in the early post-contrast phase, compatible with intrahepatic bile duct space-occupying mass. The lesion involves and infiltrates the left suprahepatic vein as well as the left branch of the portal vein and the left hepatic duct and its branches. Adenopathies in the course of the celiac trunk, measuring 17 mm, 16 mm (Figure 2).

**Figure 3:**
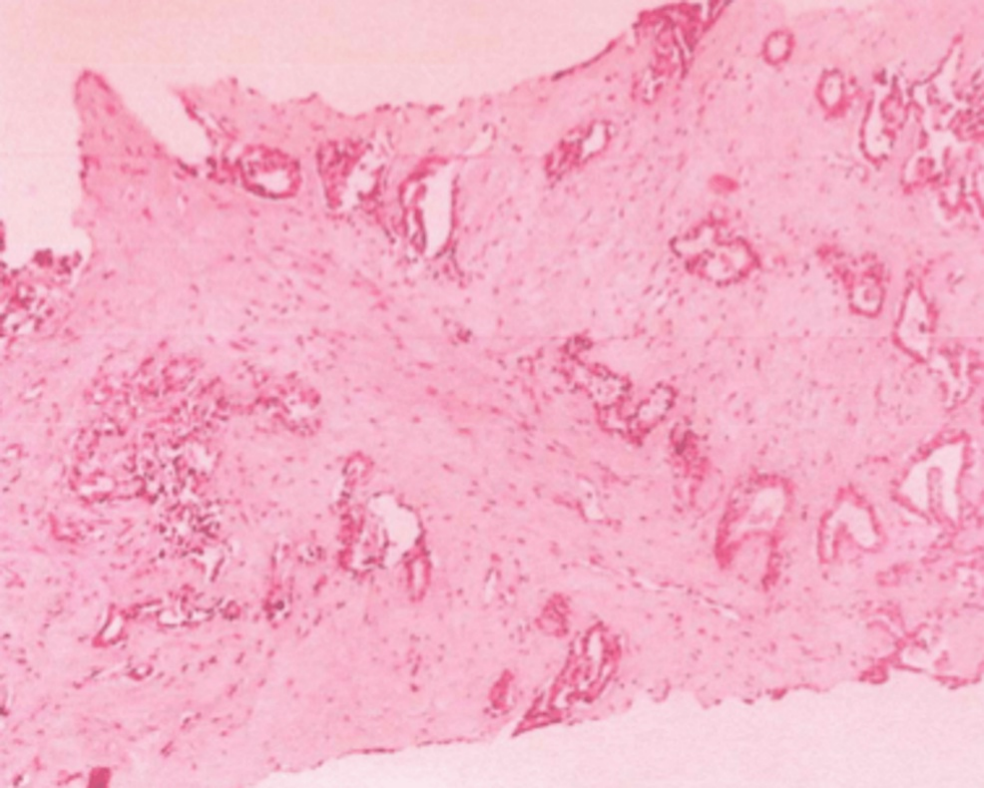
Microscopic findings of the resected liver mass exhibited rich stroma with fibrosis.

The patient underwent a CT-guided liver biopsy. Pathologic analysis showed epithelial neoplastic proliferation with irregular and coalescent pseudoglandular structures with focal intraluminal necrosis, lined by cuboidal to columnar epithelium with nuclear atypia, mitotic figures, and some subnuclear vacuoles, immersed in the stroma with marked desmoplastic fibrosis consistent with iCCA (Figure 3).

**Figure 4:**
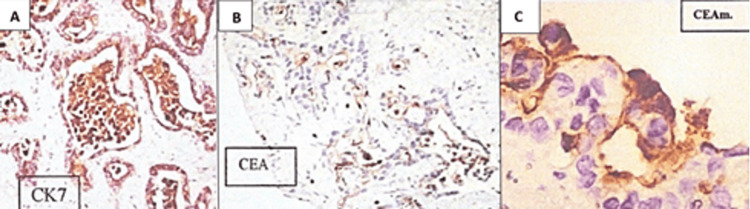
Immunohistochemical of the liver mass demonstrated that the tumor cells were positive for CK7 (A), CEA (B), and CEAm (C). CK7: cytokeratin; CEA: carcinoembryonic antigen; CEAm: carcinoembryonic antigen monoclonal

Immunohistochemical staining of the mass revealed cytokeratin (CK7) 95% positive at the edges of the neoplastic glandular epithelium, CEA and CEA monoclonal (CEAm) were 90% positive at the apical luminal edges of the neoplastic glands, negative for CD10 and CDX2. Based on these histopathological and immunohistochemical the diagnosis was consistent with well-differentiated CCA (Figure 4).

**Figure 5:**
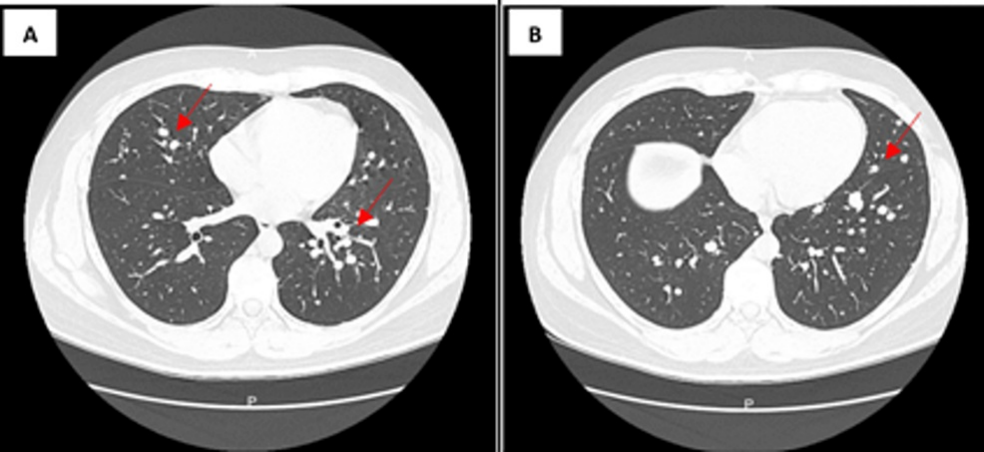
Chest CT demonstrated multiple nodules bilaterally (A) and supradiaphragmatic and paracardiac lymph nodes (B). CT: computed tomography

A high-resolution chest CT scan with and without intravenous contrast was obtained and revealed multiple, bilateral pulmonary nodules of different sizes, randomly distributed, measuring between 13 mm, 12 mm, 11 mm, some of them with post-contrast enhancement, compatible with metastatic lesions. Medial supradiaphragmatic and right paracardiac lymph nodes of 17 mm and 13 mm, no pleural effusions were observed (Figure 5).

After determining that the patient was not a surgical candidate (due to metastasis), he was started on systemic therapy with gemcitabine and cisplatin for 21 cycles. Over the course of this regimen, slight dose adjustments were necessary due to weight variations and increase in metastatic lesions.

Chemotherapy was changed to oxaliplatin, 5-fluorouracil (5-FU), and folinic acid due to persistent mass increase with a duration of four cycles after the patient presented a decompensation due to hematemesis, he underwent upper gastrointestinal endoscopy, which showed esophageal varices controlled by six-rubber-band ligation and red blood cell transfusion. Follow-up laboratories were performed while receiving gemcitabine + cisplatin and oxaliplatin, 5-fluorouracil (5-FU), and folinic acid. Results are summarized in Table 2.

**Table 2:** Laboratories while receiving treatment with gemcitabine and cisplatin (column A) and oxaliplatin, 5-fluorouracil (5-FU), and folinic acid (column B). ALT: alanine aminotransferase AST: aspartate aminotransferase GGT: gamma-glutamyl transpeptidase ALP: alkaline phosphatase

Laboratory test	Normal range	Result Column A	Result Column B
White blood cells	4.10-10.10 10x9/L	8.9 10x9/L	8.29 10x9/L
Hemoglobin	12.9-16.7 g/dL	12.5 g/dL	8.10 g/dL
Platelet count	153-328 10x9/L	108 10x9/L	86 10x9/L
ALT	21-72 U/L	27 U/L	15 U/L
AST	17-59 U/L	48 U/L	50 U/L
GGT	15-73 U/L	886 U/L	449 U/L
ALP	38.0-126.0 U/L	186 U/L	197 U/L
Albumin level	3.5-5.0 g/dL	3.8 g/dL	2.94 g/dL
Total bilirubin	0.2-1.3 mg/dL	0.57 mg/dL	0.94 mg/dL

The patient was stabilized and some days after he refused to continue with chemotherapy. He required frequent paracentesis for two months for nonmalignant ascites. The overall follow-up duration was 19 months, and the patient died 21 months after he was diagnosed.

## Discussion

CCA is a rare neoplasm with an incidence rate of 0.3/100,000 habitants per year with a predominance in males between the fifth and seventh decade of life from Western countries with an annual incidence increase of 4% [2,11]. There are multiple risk factors with strong association with iCCA development such as cholelithiasis/choledocolithiasis, non-fatty liver disease, bile duct cysts, cholangitis, chronic pancreatitis, smoking, bile duct cysts, hepatitis, cirrhosis [2,4,5,12]. However, our patient developed iCCA at the unusual age of 29 years old, as well as the absence of both personal and family risk factors.

Unfortunately, CCAs are generally diagnosed at advanced stages as a result of their non-specific clinical presentation. Signs and symptoms rely upon growth pattern, tumor location, and stage, due to this a large part of the cases are found incidentally. Although the clinical presentation, laboratory, and imaging studies serve as a guide, biopsy and immunohistochemistry are required to confirm the diagnosis [13,14]. The patient presented non-specific symptoms such as fatigue, malaise, and vomiting accompanied by altered liver tests which were complemented with CT and MRI scans which showed an occupying mass in the entirety of the left hepatic lobe and partially the right hepatic lobe these findings guided the diagnosis to neoplasm requiring biopsy and immunohistochemical staining to confirm the diagnosis.

Serological tumor biomarkers such as CA 19-9 and CEA are usually elevated in CCA, however, the diagnostic accuracy is limited, since they can be elevated in benign conditions such as cholestasis, liver injury, and other cancers. Nevertheless, CA 19-9 values> 1000 U/mL have been associated with advanced-stage or metastatic iCCA [15,16]. Even though our patient was diagnosed with metastatic iCCA the tumor biomarkers remained within normal limits.

Systemic chemotherapy is often the option for iCCA subjects with metastasis or unresectable tumor [8]. The ABC-02 study, in which patients with advanced biliary cancer were treated with the combination of gemcitabine-cisplatin and compared with patients treated with gemcitabine alone, demonstrated a higher median overall survival (11.7 vs 8.1 months, respectively; HR 0.64; 95% CI 0.52-0.8; p<0.001) in the first group, based on, it is considered the first-line chemotherapy regimen [6,8]. The ABC-06 study showed folinic acid, 5-fluorouracil, and oxaliplatin as a second line [1]. This was consistent with our patient who received a first-line regimen for 16 months but due to lack of response, it was changed to the second-line regimen.

## Conclusions

iCCA is a very aggressive type of cancer with a high mortality rate that in most cases presents symptoms when it is in an advanced stage and surgery is often no longer an alternative treatment. Despite correct diagnosis and treatment, the disease is very aggressive and the patient was already in an advanced stage with metastases in both lungs, therefore, chemotherapy was administered to prolong the time and quality of life for several months until the patient stopped responding to treatment and ultimately succumbed to the disease.
